# Characterizing a Leak in the HIV Care Cascade: Assessing Linkage Between HIV Testing and Care in Tanzania

**DOI:** 10.3389/fpubh.2019.00406

**Published:** 2020-01-30

**Authors:** Richelle Harklerode, Jim Todd, Mariken de Wit, James Beard, Mark Urassa, Richard Machemba, Bernard Maduhu, James Hargreaves, Geoffrey Somi, Brian Rice

**Affiliations:** ^1^Institute for Global Health Sciences, University of California, San Francisco, San Francisco, CA, United States; ^2^London School of Hygiene and Tropical Medicine, London, United Kingdom; ^3^National Institute for Medical Research, Mwanza, Tanzania; ^4^Magu District Council, Mwanza, Tanzania; ^5^Ministry of Health, Community Development, Gender, Elderly and Children, Dar es Salaam, Tanzania

**Keywords:** HIV, surveillance, linkage to care, Tanzania, HIV care cascade

## Abstract

**Background:** In Tanzania, HIV testing data are reported aggregately for national surveillance, making it difficult to accurately measure the extent to which newly diagnosed persons are entering care, which is a critical step of the HIV care cascade. We assess, at the individual level, linkage of newly diagnosed persons to HIV care.

**Methods:** An expanded two-part referral form was developed to include additional variables and unique identifiers. The expanded form contained a corresponding number for matching the two-parts between testing and care. Data were prospectively collected at 16 health facilities in the Magu District of Tanzania.

**Results:** The records of 1,275 unique people testing HIV positive were identified and included in our analysis. Of these, 1,200 (94.1%) responded on previous testing history, with 184 (15.3%) testing twice or more during the pilot, or having had a previous HIV positive test. Three-quarters (932; 73.1%) of persons were linked to care during the pilot timeframe. Health service provision in the facility carrying out the HIV test was the most important factor for linkage to care; poor linkage occurred in facilities where HIV care was not immediately available.

**Conclusions:** It is critical for persons newly diagnosed with HIV to be linked to care in a timely manner to maximize treatment effectiveness. Our findings show it is feasible to measure linkage to care using routinely collected data arising from an amended national HIV referral form. Our results illustrate the importance of utilizing individual-level data for measuring linkage to care, as repeat testing is common.

## Introduction

It is vital that persons who are newly diagnosed with HIV are linked to care in a timely manner to maximize treatment effectiveness and the potential of treatment as prevention (1–3). In recognition of this, in 2015 the World Health Organization (WHO) included linkage to care (the number and percentage of people living with HIV who are receiving HIV care) as one of its 10 global indicators to measure progress and drive action toward the United Nations Joint Program on AIDS (UNAIDS) 90-90-90 targets (4, 5).

Timely linkage to care remains an issue in sub-Saharan Africa, including Tanzania (6–10). The prevalence of HIV in Tanzania among adults aged 15–49 years is 5.1% (11), with an estimated 1.4 million people living with HIV (12). In 2016, Tanzania adopted the WHO guidance that antiretroviral therapy (ART) be initiated among all persons diagnosed with HIV regardless of CD4 count (12).

The National AIDS Control Program (NACP) is the coordinating unit for HIV patient monitoring and surveillance in Tanzania. To inform routine program monitoring activities, the NACP maintains aggregate HIV testing services (HTS) data, and anonymized individual level care and treatment clinic (CTC) data. A CTC number (assigned as a patient unique identifier for all treatment within a clinic) facilitates some de-duplication of these individual level data (13). With only aggregate HTS data being reported, it remains difficult to accurately numerate those newly diagnosed with HIV, or to identify persons who have been diagnosed but have not entered care.

A situational assessment was conducted in Tanzania in 2015 to evaluate the feasibility of generating individual-level longitudinal HIV data from point of diagnosis to entry into care and initiation of ART, and to leverage such data in a comprehensive strategic HIV information system, such as case surveillance (14). The WHO recommends HIV case surveillance as an integral step in securing strategic information for public health surveillance (15, 16). The situational assessment identified linkage between HIV testing and entry to care and treatment as a weakness when considering the feasibility of establishing HIV case surveillance in Tanzania (14).

To enhance NACP's capacity to track progress toward the UNAIDS 90-90-90 targets, and potentially act as a precursor for a national HIV case surveillance system, we describe a pilot project where we developed surveillance methodology to routinely collect HIV program data using a standardized referral form to assess and characterize linkage to care among persons newly testing HIV positive.

## Methods

The pilot included 16 health facilities offering HIV testing in the Magu District of the Mwanza region in Tanzania. The Magu district was chosen for the pilot as a similar expanded referral form had been utilized in one district health facility in 2008 (17). The pilot health facilities (all offering HTS) were purposely selected to ensure variation in type and size (health center, dispensary, antenatal clinic (ANC) or standalone testing site), services offered [voluntary counseling and testing (VCT), antenatal care and/or HIV care and treatment], ownership (government or private), and type of community served (rural settlement, fishing village or roadside community). The HTS offered within these facilities included VCT, ANC, laboratories [for provider-initiated testing and counseling (PITC)], and prevention of mother to child transmission of HIV (PMTCT).

Healthcare workers routinely collect data in registers on all persons testing for HIV at health facilities (18). We expanded the standard paper-based NACP referral form into a two-part paper form that included additional variables to be collected from all clients testing HIV positive. In this two-part form, health workers completed 19 questions on both parts of the form, with an additional four questions on the part retained by the health facility. These four question were: other names used by patient, whether they had previously received an HIV positive test result and, if they had, time and place of that test (Table 1). The “other names used” variable was included to facilitate patient follow-up and case matching. Previous positive test was introduced to identify potential duplicate records arising from repeat testing. The expanded form also contained a corresponding form number for matching the two-parts between testing and care.

**Table 1:** Data availability for the expanded referral form.

	**HIV testing registers**	**Routine aggregate HIV testing data**	**Standard NACP referral form**	**Routine clinic data from HIV care**	**Additional data requested through pilot**
**Patient demographics**					
Patient first name	X	–	X	X	–
Patient second name	X	–	X	X	–
Patient last name	X	–	X	X	–
Other names used by patient	–	–	–	–	X
Age in years	X	X (categorized)	X	X	–
Year of birth	X	–	X	X	–
Sex	X	X	X	X	–
**Reporting facility and HIV diagnosis**					
HTS facility name	X	X	X	X	–
HTS type	–	X	–	X	–
Visit (test) date	X	–	X	X	–
Clinic referred to	X	–	X	X	–
Name of referring nurse	X	–	X	–	–
**Patient location information**					
District	X	–	–	X	–
Ward	X	–	–	X	–
Village	X	–	–	X	–
Sub-village	X	–	–	X	–
Ten cell leader^*^		–	–	X	–
**Patient-specific identifiers**					
Client VCT/ANC number	X	–	–	X	–
Name someone else in household	–	–	–	X	–
Phone number	–	–	–	X	–
**Clinical information**					
Pregnant	X	X	X	X	–
Tested HIV positive before	X	–	–	X	X
When tested HIV positive before	–	–	–	X	X
Where tested HIV positive before	–	–	–	X	X
**HIV care**					
CTC file number	–	–	–	X	–
CTC patient number	–	–	X	X	–
Date first seen	–	–	X	X	–

* *Ten-cell leader is an appointed number of households (originally 10 but often now higher), reporting to village, and sub-village authorities*.

We trained facility staff to collect pilot data prospectively on the referral form from 1st January 2017 to 31st December 2017. Facility staff were provided a small stipend (2,000 TSH ~US$1) for correct completion of each part of the form. Facility staff were instructed to complete both parts of the form; the first part of the form, with the extra questions, was retained, with the second part given to the client who was then instructed to provide it to the CTC at first attendance. Staff at the CTC were instructed to complete the bottom portion of the form (the section on HIV care) when the patient enrolled in HIV-related care.

A pilot fieldworker visited each participating facility once a month to gather completed referral forms and to review completeness. Data from the forms were entered into an Access database. The database was developed by the research team and included inbuilt validation rules to minimize data entry errors (for example, checking the age against year of birth, and the sex and age against pregnancy status), and to facilitate the matching of corresponding form numbers on the two-parts. Records with missing key variables (name, referral form number, sex, or age) were excluded from data analysis. De-duplication of cases was performed using personal identifying information, either using the three given names, sex, and age of the participant, or by matching one of the names, age, and sex with the same residence for exact matches.

Linkage to care was assessed through matching corresponding parts of the two-part referral form based on the form number and review of patient name. For persons who presented at the CTC without their referral form, a probabilistic match, similar to one used in a South Africa study, based on a close match of three out of four variables, name, sex, age, and/or residence, was used to link the CTC attendance to the referral form (19). Our definition of linkage to care is therefore based on evidence (referral form or probabilistic matching) of having presented at a CTC at least once. Time elapsed between testing and linkage to care was measured according to a person's date of HIV diagnosis (initial diagnosis) and date first seen in CTC (as presented on their two-part referral form). To allow follow-up time for linkage to care of those diagnosed toward the end of 2017, the last extraction of data occurred on 24th March 2018.

To assess ART use among persons linked to care, data from participating CTCs were extracted from electronic systems or the paper-based CTC form. A persons CTC number (as presented on their referral form) was used to match data to their CTC record. The records of persons linked to care for whom key variable information was missing, or for whom the CTC file could not be identified, were excluded from this analysis. In receipt of ART was defined as being on ART at any point during the follow-up period.

Ethical approval was obtained from the Tanzanian National Research Ethics Committee (NatREC Ref NIMR/HQ/R.8a/Vol.IX/2097 extended on 9th March 2018 with NIMR/HQ/R.8c/Vol.II/961) and the London School of Hygiene and Tropical Medicine (#11844). Data used in these analyses were password protected and all study coordinators, data abstractors, and analysts signed a confidentiality form.

Analysis was performed in Stata 14 (Stata Corp., USA). Frequencies and cross tabulations were conducted, as was logistic regression to obtain odds ratios and 95% confidence intervals for factors associated with linkage to care. P-values are reported to show statistical compatibility of the data with the null hypothesis.

## Results

Between 1st January 2017 and 31st December 2017, 1,312 persons were diagnosed with HIV across the 16 pilot facilities and had their information captured. Data quality activities resulted in the records of 32 (2.4%) people being further investigated. Of these, 13 were excluded from the analysis due to missing sex or age. The records of an additional 24 (1.8%) people were identified as having a duplicate record as a result of repeat testing within the follow-up period. Of these 24, five re-tested within the same facility and 19 re-tested at a different pilot facility. In total, the records of 1,275 unique people testing HIV positive were identified and included in our analysis.

Completeness for most variables exceeded 98%. The exceptions were, name of a person who lived in the same house (1,119; 87.8%), telephone number (708; 55.5%), and the additional question on previous positive HIV test (1,200; 94.1%).

### Demographic and Testing Characteristics

Table 2 presents the demographic and testing characteristics. The male to female ratio was 1:1.4, with pregnant women accounting for 21.4% (159) of female participants. Median age at diagnosis was 32 years. A quarter of people (298; 23.4%) came from Kisesa ward (where the main health center in the Magu District is situated), with three other wards contributing 10% each (129 in Bujashi, 137 in Nyanguge, and 127 in Kitongo). The two most common diagnosing HTC types were PITC (556; 43.6%) and VCT (362; 28.4%). Interestingly, Table 2 shows almost one in five men testing HIV positive to have done so through partner testing in ANC/PMTCT.

**Table 2:** Demographic, testing, and clinical characteristics of persons testing HIV positive^*^.

			**Tested HIV positive previously (*****n*** **= 1,200)**			
	**HIV positive test (*****n*** **= 1,275)** ***n*** **(%)**		**No (*****n*** **= 1,040 new diagnosis)** ***n*** **(%)**		**Yes (*****n*** **= 160 repeat diagnosis)** ***n*** **(%)**	
	**Female**	**Male**	**Female**	**Male**	**Female**	**Male**
**Total**	**743**	**532**	**591**	**449**	**109**	**51**
**Age at diagnosis (years)**						
0–4 years	13 (1.8)	20 (3.8)	11 (1.9)	18 (4.0)	1–5	1–5
5–14 years	20 (2.7)	17 (3.2)	18 (3.0)	14 (3.1)	0	1–5
15–19 years	26 (3.5)	1–5	21 (3.6)	5 (1.1)	1–5	0
20–24 years	145 (19.5)	29 (5.5)	117 (19.8)	25 (5.6)	23 (21.1)	1–5
25–49 years	477 (64.2)	377 (70.9)	373 (63.1)	317 (70.6)	74 (67.9)	38 (74.5)
50+ years	62 (8.3)	84 (15.8)	51 (8.6)	70 (15.6)	6 (5.5)	9 (17.6)
**Ward of residence**						
Kisesa	174 (23.4)	124 (23.3)	138 (23.4)	102 (22.7)	25 (22.9)	13 (25.5)
Nyanguge	92 (12.4)	45 (8.5)	68 (11.5)	39 (8.7)	21 (19.3)	6 (11.8)
Bujashi	73 (9.8)	56 (10.5)	59 (10.0)	52 (11.6)	10 (9.2)	1–5
Kitongo	69 (9.3)	58 (10.9)	65 (11.0)	55 (12.2)	0	0
Other wards	332 (44.7)	249 (46.8)	258 (43.7)	201 (44.8)	53 (48.6)	29 (56.9)
Missing	3 (0.4)	0	3 (0.5)	0	0	0
**Pregnant**						
Yes	159 (21.4)	—	126 (21.3)	—	28 (25.7)	—
No	563 (75.8)	—	450 (76.1)	—	81 (74.3)	—
Don't know	12 (1.6)	—	11 (1.9)	—	0	—
Missing	9 (1.2)		4 (0.7)		0	
**HTC type**						
VCT	211 (28.4)	151 (28.4)	162 (27.4)	130 (29.0)	41 (37.6)	20 (39.2)
ANC/PMTCT	168 (22.6)	96 (18.0)	134 (22.7)	77 (17.1)	24 (22.0)	11 (21.6)
PITC	314 (42.3)	242 (45.5)	251 (42.5)	201 (44.8)	38 (34.9)	18 (35.3)
Other	49 (6.6)	42 (7.9)	43 (7.3)	40 (8.9)	6 (5.5)	1–5
Missing	1 (0.1)	1 (0.2)	1 (0.2)	1 (0.2)	0	0
**Linked to care**						
Yes	547 (73.6)	385 (73.4)	436 (73.8)	332 (73.9)	83 (76.2)	34 (66.7)
No	196 (26.4)	147 (27.6)	155 (26.2)	117 (26.1)	26 (23.8)	17 (33.3)
In receipt of ART (persons linked to care with a matched CTC record)^**^						
Yes	—	—	382 (93.4)	271 (90.3)	67 (95.7)	36 (94.7)
No	—	—	27 (6.6)	29 (9.7)	3 (4.3)	2 (5.3)

* *Where cell counts <5 masked a range was given to protect anonymity of participants*.

** *N = 817 [932 people were linked to care (as identified through matching the two parts of the referral form or probabilistic matching); of these it was possible to match the CTC data to the HTC referral testing records for 818; of these 818, 1 had missing ART status]*.

### Previous HIV Positive Test

The characteristics of the 1,200 people reporting previous test history are presented in Table 2. In total, 160 (13.3%) people reported having had a previous HIV positive test result. In addition, 24 people tested positive twice during the pilot period, resulting in a total of 184 (15.3%) people having more than one HIV positive test. Females were more likely to report a previous positive test (15.5%; 109) compared to males (10.2%; 51) (*p* = 0.008).

One in five (28/153; 18.3%) previously tested within 1 year of their current test, with four in five (122/153; 79.7%) previously testing within 4 years. Three quarters (120/158; 75.9%) previously tested positive in a site different to the one they presented at during the pilot.

### Linkage to Care

The 1,275 HIV positive people were asked to which facility they wished to be referred (all but two responded). Almost all people (1,223; 95.9%) chose a referral facility included in the pilot, with 80.0% (1,019/1,273) asking to be referred to the CTC situated in the facility in which they had just tested HIV positive.

In total, 932 (73.1%) of people testing HIV positive were successfully linked to care (as identified by a referral form or probabilistic matching) during the pilot period. The majority of persons linked to care were seen at the facility to which they were referred (880; 94.4%), and/or were first seen the same day they received their test result (756; 81.1%). Among those not seen the same day, median time from diagnosis to entering care was 3 days (IQR 2–7 days).

Forty (4.3%) of the 932 people linked to care did not provide their referral form when first attending. Of these, all attended on a different date to that on which they were referred, and 11 (27.5%) attended a different facility to the one in which they were tested.

Table 3 presents rates of, and factors associated with, linkage between testing, and care. In univariate analysis, ward of residence, pregnancy status, and testing facility services type were associated with linkage between testing and care (all *p* < 0.05). These associations all persisted after multivariate adjustment. In summary, the following groups were those most likely to be linked to care: living within the Magu district or catchment wards; currently pregnant; received HIV positive test in a dispensary facility that offers CTC services.

**Table 3:** Factors associated with linkage from testing to care.

**Characteristic**	**Positive tests**	**Linked to care *n* (%)**	**OR (95% CI)**	*P*-value^**^	Adjusted OR (95% CI)^***^	*P*-value^**^
**Sex**						
Male^*^	532	385 (72%)	1	0.62		
Female	743	547 (74%)	1.07 (0.83–1.37)			
**Age at most recent diagnosis**						
<15 years	70	57 (81%)	1.59 (0.86–2.96)	0.12		
15–24 years	205	142 (69%)	0.82 (0.59–1.14)			
25+ years^*^	1,000	733 (73%)	1			
**District of residence**						
Catchment wards^*^^$^	895	667 (75%)	1	*0.0004*	1	*0.03*
Magu district	267	201 (75%)	1.04 (0.76–1.43)		1.30 (0.90–1.86)	
Other district	113	64 (57%)	0.45 (0.30–0.67)		0.64 (0.40–1.01)	
**Pregnancy status**						
Male^*^	532	385 (72%)	1	*0.0006*	1	* <0.0001*
Yes	159	134 (84%)	2.05 (1.28–3.27)		3.12 (1.83–5.33)	
No	563	402 (71%)	0.95 (0.73–1.24)		0.93 (0.70–1.25)	
Don't know	12	5 (42%)	0.27 (0.08–0.87)		0.46 (0.12–1.86)	
**Previous HIV positive test**						
No^*^	1,040	768 (74%)	1	0.37		
Yes ≥5 years ago	25	21 (84%)	1.86 (0.63–5.46)			
Yes <5 years ago	128	91 (71%)	0.87 (0.58–1.31)			
**HTC type**						
VCT	362	276 (76%)	1.33 (0.98–1.80)	0.23		
ANC/PMTCT	264	191 (72%)	1.08 (0.78–1.50)			
PITC^*^	556	393 (71%)	1			
Other	91	70 (77%)	1.38 (0.82–2.33)			
**Type of testing facility**						
Health center with CTC^*^	658	508 (77%)	1	* <0.0001*	1	* <0.0001*
Dispensary with CTC	402	348 (87%)	1.90 (1.35–2.67)		1.72 (1.22–2.44)	
ANC/PMTCT (no CTC)	134	58 (43%)	0.22 (0.15–0.33)		0.16 (1.11–0.25)	
HTC only (no CTC)	78	15 (19%)	0.07 (0.04–0.13)		0.07 (0.04–1.13)	

*Numbers are as among those providing a response*.

* *Reference group*.

** *Significant associations at the 95% level are shown in italics*.

*** *Variables identified in univariate analysis as being associated with linkage to care included in multivariate model*.

$ *Catchment areas for the 16 health facilities; dispensaries serve a village within a ward whereas health centers serve a ward*.

Of the 932 patients liked to care, we were able to find a CTC record for 909 (97.5%, 818 through matched CTC number and names on referral form and 91 through probabilistic matching), confirming they were not only linked to, but also enrolled in, care. Of these 909 people, 756 (83.2%) initiated ART (representing 59.3% of total positives), with the remaining 153 (16.8%) being seen only once at the facility. The majority (489; 64.7%) of persons who initiated ART did so on their first visit to the clinic. Among those remaining, median time from entry to care and ART initiation was seven days (IQR 6–13 days).

Based on information on previous positive test, 108 (67.5%) people were determined to have previously attended a CTC. Three (1.9%) were determined to have transferred from another CTC having already been in receipt of ART, and eight (5.0%) had previously been in receipt of ART (all more than a year previously).

## Discussion

The pilot findings support the use of an expanded referral form for assessing, at the individual-level, linkage of newly diagnosed persons to HIV care. The system is simple and replicable, providing quality data (variable completeness typically >90%) in a high HIV burden, resource-poor setting. The pilot demonstrated that the paper-based referral system facilitated the collection of key variables necessary to describe and track the demographic and clinical characteristics of persons diagnosed and receiving HIV care, to de-duplicate records in a systematic manner, and to enhance our understanding of the HIV epidemic.

Three in four people diagnosed with HIV subsequently enrolled in care; with the overwhelming majority doing so the same day they tested positive, and at the facility to which they were referred. Factors most strongly associated with being linked to care were being currently pregnant, and being diagnosed positive in a dispensary facility that offers CTC services. Whereas, eight in10 pregnant women diagnosed with HIV were subsequently linked to care (highlighting there is still room for improvement within this highly important group) it is of interest that no significant association between HTC type (which includes ANC and PMTCT) and linkage to care was observed. A possible explanation for this, is that testing in ANC/PMTCT included not only women but also a sizeable number of male partners.

The association with the type of CTC services being offered at the testing facility suggests structural barriers to access of care, which can only be resolved through enhanced service planning. Other studies conducted in Tanzania have found similar associations. A study conducted in the Mbeya region found that people testing positive at a facility with a CTC, were 78% more likely to link to care compared to people testing at mobile/outreach sites (7). Another study in the Tanga region found a significant association between early entry in care and point of diagnosis, level of education and CD4 count (9). A study in the Kilimanjaro region showed that people testing in a community VCT facility were twice as likely to delay care compared to people testing in a hospital outpatient department (10).

Our finding that approximately a quarter of cases did not link to care during the pilot, is similar to the findings of other studies in Tanzania. The study in the Kilimanjaro region found that only 70% had presented to for HIV care within 6 months after receiving a positive HIV test result (10), and in the Mbeya region study, 78% linked to care within 6 months (7). Linkage to care rates vary throughout sub-Saharan Africa. In Kenya, 88% of adults linked to care within three months (20); a field assessment in Mozambique showed 67% linked to care (21); a retrospective cohort study in Cape Town, South Africa identified linkage to care as 63% (22); in Lesotho, a study found linkage to care at a facility to be 43% versus 69% if ART was initiated at home (23); and a systematic review showed linkage to care of newly diagnosed with HIV in sub-Saharan Africa ranged from 10–79% (24). As the expanded referral form includes location information it will be possible for facilities to intervene to locate and engage with persons not immediately linking to care; this important intervention was not assessed during the pilot.

Approximately 15% of people included in the pilot were found to have tested HIV positive more than once, of these 70% were previously linked to care. This finding highlights the importance of obtaining individual-level data on previous HIV test results, and potentially, a need to consider adjusting for such duplicate records in prevalence estimates based on standard aggregate reporting (wider geographically based estimates would be required to inform such an adjustment).

Introducing a form number on both parts of a two-part referral form assisted with matching a persons records between testing and care. We should note, however, that the form number does not help match cases that retest for HIV. De-duplication of these records was conducted, using additional identifiers available on the referral form. It is possible that the provision of a small stipend resulted in high variable completeness which, in turn, contributed to the ability to effectively match records. This process would be more difficult on a larger scale; an algorithm would need to be tested on a larger dataset, as is used in other countries without a national unique identifier (25–27).

The NACP already has a standard referral form and since the majority of data are already routinely collected, the work effort for facility staff to continue to complete the two-part form would be minimal. The additional cost of data entry of the referral forms could also be seen as minimal when compared to the enhanced availability and accuracy of the data. The pilot utilized field staff to gather forms from the facility on a monthly basis. This was feasible at the scale the pilot was conducted but could prove to be a human resource burden at national scale. Additionally, the pilot utilized an Access database, for implementation at national level, data entry and analysis would need to be incorporated into the existing national system. An assessment should be undertaken on alternate strategies for data collection and entry.

Due to practical limitations, the pilot was conducted in only a portion of the facilities in one district. As such, linkage to care could have occurred at a facility outside of the pilot. No hospitals were included, therefore the pilot does not assess the burden the linkage system would have on a larger facility which has more cases of HIV. Despite these limitations, the pilot provides valuable information that is not available elsewhere and that can be used to determine the way forward for linking patients to HIV care and identifying potential duplicate records.

Case matching and de-duplication are of vital importance for accurately assessing the 90-90-90 targets and HIV care cascade, and for moving toward HIV case surveillance. The ability to distinguish between new and previously reported cases increases the quality of the data, and of analyses using these data. The 2017–2022 HIV Strategic Plan in Tanzania calls for strengthening linkage mechanism, and notes that a challenge to achieving this is the lack of reliable referral data (28). To utilize de-duplicated data to monitor the HIV epidemic and ensure linkage from testing to care timely manner, we recommend a two-part referral form be used for all HIV diagnoses. A new initiative by NACP to collect all HTC data in the CTC electronic database may also provide a better understanding of HTC and CTC attendance, although will require rigorous evaluation.

## Data Availability Statement

The datasets for this article contain protected health information and are not publicly available. Requests to access the datasets should be directed to Jim Todd, Jim.Todd@LSHTM.ac.uk.

## Author Contributions

The concept for the study was developed by JT, MU, BR, and RH. RM coordinated data collection. RH, MW, JT, JB, JH, RM, and BR conducted data analysis and/or data interpretation. All authors read the manuscript, provided feedback, and approved the final version.

### Conflict of Interest

The authors declare that the research was conducted in the absence of any commercial or financial relationships that could be construed as a potential conflict of interest.
